# Morbidity and Mortality in Patients over 90 Years of Age Following Posterior Stabilization for Acute Traumatic Odontoid Type II Fractures: A Retrospective Study with a Mean Follow-Up of Three Years

**DOI:** 10.3390/jcm10173780

**Published:** 2021-08-24

**Authors:** Mohammed Issa, Karl L. Kiening, Andreas W. Unterberg, Moritz Scherer, Alexander Younsi, Stepan Fedorko, Rod J. Oskouian, Jens R. Chapman, Basem Ishak

**Affiliations:** 1 Department of Neurosurgery, Heidelberg University Hospital, 69120 Heidelberg, Germany; mohammed.issa@med.uni-heidelberg.de (M.I.); karl.kiening@med.uni-heidelberg.de (K.L.K.); andreas.unterberg@med.uni-heidelberg.de (A.W.U.); moritz.scherer@med.uni-heidelberg.de (M.S.); alexander.younsi@med.uni-heidelberg.de (A.Y.); stepan.fedorko@med.uni-heidelberg.de (S.F.); 2 Complex Spine Surgery, Swedish Neuroscience Institute, Seattle, WA 98122, USA; rod.oskouian@swedish.org (R.J.O.); jens.chapman@swedish.org (J.R.C.)

**Keywords:** odontoid type II fractures, nonagenarians, elderly, posterior stabilization, mortality, complications, comorbidity

## Abstract

Odontoid type II fractures represent the most common cervical spine injuries in the elderly. The decision for surgical treatment in very elderly patients is still controversial. The aim of this study was to assess morbidity and mortality in patients over 90 years of age undergoing CT-guided posterior stabilization for unstable odontoid type II fractures. A total of 15 patients with an acute traumatic odontoid type II fracture who received surgical treatment for unstable odontoid type II fractures were retrospectively analyzed. Complications, morbidity, and mortality as well as length of ICU and hospital stay were determined. Clinical follow-up evaluation was based on outpatient presentation and information from family members and general practitioners. Finally, we conducted a comparison of complications rates between patients over 90 years of age and patients between 65 and 89 years old with a type II odontoid fracture after CT-guided posterior stabilization in our institution. The mean age was 91.4 years. Patients were predominately female (87%). In-hospital deaths did not occur. The average length of the hospital stay was 13.4 days and 1.9 days for the ICU. Blood transfusion was necessary in two patients (13%). Two patients (13%) developed urinary tract infection, one patient (7%) a delirium, and another epistaxis (7%). One patient (7%) developed pneumonic sepsis and fully recovered within several weeks. The mean follow-up was 36 months (range 9–72 months). Implant-related complications developed in one patient (7%). Five patients died during the follow-up period, with an average time to death of 26.6 months. Postoperative bracing was not needed in any of the patients. Posterior stabilization of unstable odontoid fractures type II using CT-guided navigation in patients over 90 years of age is a safe and effective procedure with low complications and mortality rates.

## 1. Introduction

**Figure 1:**
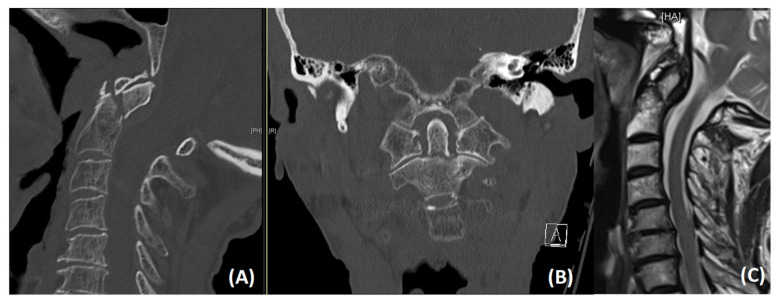
Sagittal (**A**) and coronal (**B**) CT images showing an unstable dislocated odontoid type II fracture in a 90-year old male patient. T2-weighted sagittal MR image (**C**) illustrating a slight anterior spinal cord compression.

Recent advances in surgical treatment and anesthesiological management have increased the demand for spinal surgery to achieve a higher quality of life [1,2,3]. In older individuals, odontoid type II fractures (Figure 1) represent the most common, acute, traumatic cervical spine injuries, which are most likely due to low-velocity mechanisms after simple falls from a standing position or sitting height [4]. Here, the body’s ability to absorb the energy of trauma is significantly reduced due to osteoporosis and progressive degenerative changes [5,6].

Although the optimal management strategy for elderly patients with odontoid type II fractures is still quite ambiguous, clinical, radiological, and functional outcomes after posterior stabilization have recently been shown to be favorable in numerous studies [7,8,9]. Despite those results, a hesitancy to treat remains due to poor bone quality and medical comorbidities that can contribute to an increased surgical risk [10].

On the other hand, numerous patient series and an AOSpine-based prospective multicenter study have shown that conservative treatment with halo vest immobilization results in significantly higher rates of non-union and morbidity, which carry an increased risk of aspiration pneumonia and reduced ability of swallowing [11,12,13].

Striking the right balance between these options poses a difficult challenge, especially when very elderly and often multimorbid patients are involved.

Larger studies focusing on geriatric patients over 80 years of age are scarce and studies exclusively addressing surgical treatment in patients over 90 years of age are nonexistent. The fact that surgeons are now confronted with an aging society makes it reasonable to offer posterior stabilization surgery not only to elderly but also to very elderly patients with unstable odontoid type II fractures, as this has already been proven to be a safe and effective treatment option, despite diminished cardiopulmonary function [7,8,13,14]. On the other hand, the heterogeneity of study methodologies reported in the literature should be considered [15].

For the aforementioned reasons and owing to more than 10 years of experience of a favorable outcome in performing posterior stabilization in elderly patients with acute unstable odontoid type II fractures, our institution favors surgery via a posterior approach in elderly patients, especially in octo- and nonagenarians.

To the best of our knowledge, this is the first study to analyze morbidity and mortality in patients over 90 years of age with a special emphasis on peri- and postoperative complications and intermediate-term results.

## 2. Materials and Methods

### 2.1. Study Design and Patient Population

We conducted a retrospective, single-center study based on a prospectively kept database. Following local ethics committee approval (Study ID: S-115/2015) and obtaining informed consent from all patients or their relatives/authorized caregivers, we included 15 patients ≥ 90 years who had received posterior stabilization surgery for acute, unstable, odontoid type II fractures between January 2010 and December 2019 at our level-I trauma center. The average follow-up (FU) was 36 months (range 9–72 months). A detailed description of inclusion and exclusion criteria is presented in Table 1.

The diagnosis of an acute odontoid fracture was based on a thin-slice computed tomography (CT) scan. CT angiography was added to determine anatomical abnormalities or kinking in the course of the vertebral artery before surgery. Furthermore, magnetic resonance imaging (MRI) of the cervical spine was conducted to assess the integrity of the spinal ligaments and the spinal cord.

### 2.2. Surgical Technique

**Figure 2:**
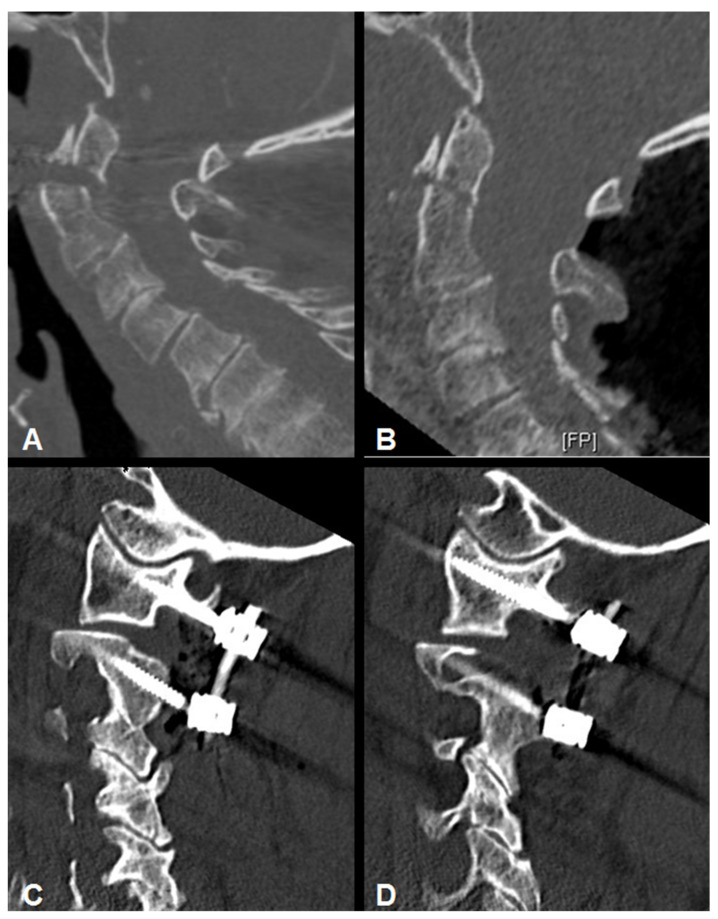
(**A**) Sagittal CT-image of a 91-year old male with an unstable odontoid type II fracture. (**B**) Intraoperative Sagittal CT data set for spinal navigation showed complete fracture reduction. Postoperative sagittal CT reconstruction after atlanto-axial stabilization using the posterior arch C1 lateral mass (PALM) technique (**C**,**D**).

Posterior cervical fusion was performed in all 15 patients using a modified Goel–Harms technique with a so-called posterior arch C1 lateral mass (PALM) screw [16,17,18]. Not only are there fewer cortical breaches, but bleeding from the epidural venous plexus and manipulation of the C2 nerve root with a potential risk of occipital neuralgia can also be avoided when using an entry point through the posterior arch of C1. Usually, C2 pedicle screws were inserted according to Harms’ technique [19]. In 3 cases with narrow C2 pedicles (less than 4 mm in diameter), C2 isthmic screws were placed, and the construct was extended to C3 (2 patients) or C4 (1 patient) to enhance biomechanical stability. All screws in all patients were placed under intraoperative CT-guided point-to-point navigation to achieve maximum safety and screw accuracy [20]. For this study, a polyaxial titanium alloy screw system (Mountaineer OCT System, DepuySynthes, Warsaw, IN, USA) was used. The fusion material was placed after exposing the cancellous bone by using a navigated high-speed drill (Figure 2). Patients were not required to wear a cervical orthosis after surgery.

### 2.3. Outcome Parameters

Numerous clinical parameters, such as body mass index (BMI), American Society of Anesthesiologists (ASA) score, hospital stay, intensive care unit (ICU) stay, intraoperative blood loss, and necessity of blood transfusion were assessed. Intra- and postoperative complications as well as mortality were recorded. Comorbidities were assessed preoperatively based on the age-adjusted Charlson Comorbidity Index (AACCI) [21,22]. The AACCI score was calculated for each patient to classify comorbidity and grouped as having either no comorbidity (AACCI = 0), minimal comorbidity (AACCI = 1–2), moderate comorbidity (AACCI = 3–5), or severe comorbidity (AACCI > 6).

Routine clinical and radiological follow-up examinations were routinely performed before discharge as well as 3 months after surgery and at final follow-up, which varied between 9 and 72 months postoperatively. Standard X-ray and CT scans in anteroposterior and lateral view were performed to evaluate screw position and fusion rate. Osseous fusion was defined as the presence of a trabecular bony bridging, assessed by an independent radiologist.

To measure the effect of the rehabilitation, the Barthel Index was used to assess independence in activities of daily life before surgery and after rehabilitation [23].

At the final follow-up, patients/authorized caregivers or their family doctors were contacted to answer a questionnaire regarding neurological status, morbidity, mortality, and current Barthel Index.

### 2.4. Comparison of Complication Rates with Patients under 90 Years of Age

Between February 2010 and December 2019, a total of 72 patients aged between 65 and 89 years old (mean age 78.8 years old, 55.4% female) with a type II odontoid fracture after CT-guided posterior stabilization were selected as reference group to compare complication rates within different age groups.

### 2.5. Statistical Analysis

Normal distribution was tested using the Shapiro–Wilk test. Continuous variables were reported as mean ± standard deviation and categorical variables as frequencies and percentages. The t-test was used for intergroup comparisons for continuous variables and the Mann–Whitney-U test was used for intergroup comparisons for categorical variables. A *p*-value of less than 0.05 was used to indicate a statistically significant difference. Statistical analyses were performed using SPSS 25 (IBM-Corp, Armonk, NY, USA).

## 3. Results

### 3.1. Patient Characteristics

A total of 15 patients met the inclusion criteria and were included in this study. The study population comprised 2 men (13%) and 13 women (87%), with a mean BMI of 23.1 ± 3 kg/m^2^, representing normal weight status. The median age at the time of surgery was 91.4 ± 2 years (range 90–96 years).

Preoperative narcotic-relevant ASA scoring revealed 3 patients (20%) with an ASA 2 score and 12 patients (80%) with an ASA 3 score; thus, 80% of patients were at increased risk of perioperative complications. All patients included in our study presented with at least 3 comorbidities, with a mean AACCI value of 9.1 ± 3.4 for the entire cohort, indicating a severe comorbidity status (Table 2). Comorbidities are summarized in Table 3.

The average length of hospital stay was 13.4 days and 1.9 days for ICU. All patients remained neurologically intact postoperatively and could be mobilized on day one after surgery under the supervision of a physiotherapist.

### 3.2. Mortality

In-hospital mortality was 0%. A total of five patients (33%) died during the follow-up period. Two patients died 12 and 35 months after surgery for unknown reasons. One patient died 56 months after surgery due to acute decompensated heart failure. One patient died 21 months after surgery of hepatocellular carcinoma that was diagnosed at a later stage. The fifth patient died 9 months after surgery as a result of a heart attack. All the deceased patients had at least five comorbidities. The average time to death was 26.6 months (range 9–56 months).

### 3.3. Clinical and Functional Characteristics

**Figure 3:**
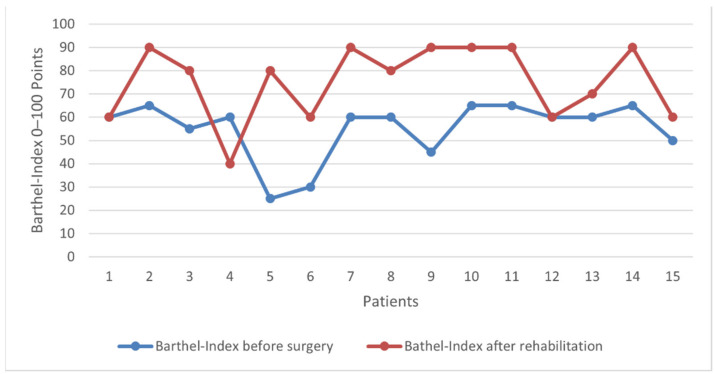
Preoperative and postoperative (after completion of the rehabilitation) improvement of the general and health care status, measured with the Barthel Index for each included patient.

After discharge from the hospital, all 15 patients completed a 14-day geriatric rehabilitation, with the goal of returning the patient to a preinjury quality of life. Before surgery, the mean Barthel Index was 55 points (severe dependence) and improved significantly to 75 points after rehabilitation, indicating moderate dependence (*p* < 0.05), Figure 3. Thirteen patients (86.7%) stated that they were independent before trauma. Postoperative bracing was not needed in any of the patients, effectively avoiding the risk of complications such as aspiration pneumonia.

### 3.4. Radiographic Results

Intraoperative and postoperative X-ray and CT imaging revealed correct screw placement. Proper alignment of the atlantoaxial spine could be achieved in all cases. No signs of screw loosening or migration were seen in the radiological routine control between 3 and 6 months postoperatively. One C2 screw was penetrating the transverse foramen with a close relationship to the vertebral artery. However, this patient remained without any symptoms. Postoperative CT angiography did not reveal any vertebral artery stenosis or flow disturbance. Fusion could be achieved in all patients by clearly visible, continuous bony trabeculation as evaluated by radiographic imaging at the final FU.

### 3.5. Complications and Perioperative Course

No intraoperative complications developed. Blood transfusion during surgery was necessary in 2 patients due to a dual antiplatelet therapy. The mean intraoperative blood loss was 243.3 mL for all patients. No patient required surgical revision during the early postoperative phase. Fourteen out of 15 patients could be mobilized one day after surgery. One patient developed sepsis and could not be mobilized immediately after surgery.

One major complication developed (Table 4). One patient developed a pneumonic sepsis caused by inferior lobe pneumonia after surgery. Therefore, the overall major complication rate was 7%.

Minor complications, which occurred in 27% of cases, could be treated successfully using medication (Table 4). No patient suffered permanent complications.

### 3.6. Comparison of Complication Rates with Patients under 90 Years of Age

A total of 72 patients between 65 and 89 years old (mean age 78.8 years old, 55.4% female) with an odontoid type II fracture after CT-guided posterior stabilization were analyzed. The preoperative ASA score, postoperative ICU stay, and 90-day-mortality did not differ significantly between the two groups (*p* < 0.05). Nine patients (12.5%) suffered from minor complications, such as conservative treated wound healing disorder (*n* = 2), urinary tract infection (*n*= 3), and pneumonia (*n* = 4). Five patients (6.9%) developed postoperative major complications such as cardiopulmonary decompensation. On the other hand, only four patients (27%) over 90 years of age showed minor complications and one patient (6.7%) had major complications (Table 4 and Table 5).

## 4. Discussion

Fractures of the C2 vertebra with predominantly odontoid type II fractures constitute the most common cervical spine injury in the elderly, with a rising incidence during the last two decades in Western countries [24,25,26,27]. Surgical fixation of unstable odontoid type II fractures in the elderly, who often present with multiple comorbidities and decreased cardiopulmonary reserves, is still a subject of debate due to high rates of complications, morbidity, and mortality [28]. On the other hand, conservative treatment with a halo vest or rigid cervical collar also correlates with a high morbidity and mortality as a result of cardiac arrest, pneumonia, and pulmonary embolism [29]. Nonsurgical treatment strategies for odontoid type II fractures have recently revealed a complication rate in up to 66% and an early death rate in up to 42% [11,30,31,32,33,34]. Furthermore, nonunion rates have been described in up to 85% due to inadequate reduction or insufficient stabilization and fracture displacement [35,36,37,38]. However, recent studies have reported a favorable clinical, functional, and radiological outcome of surgical treatment in very elderly patients with unstable odontoid type II fractures with low rates of complications and mortality [8,13,39]. Therefore, spine surgeons worldwide opt for early surgery in this cohort.

To the best of our knowledge, this current study represents the first one to address intermediate-term outcome in surgically treated nonagenarian patients with unstable odontoid type II fractures. Our results demonstrate that posterior stabilization in nonagenarian patients is a safe and effective procedure when using intraoperative CT navigation guidance. The mean length of hospital stay in our study was 13.4 days with an average ICU stay of 1.9 days, which is relatively low in comparison to previously published studies [13,16,39,40] and highlights the importance of an ICU in this often multimorbid patient cohort. One-third of the patients developed minor complications with full recovery over time. The rate of major complications was surprisingly low at 7%, which is comparable to the current literature addressing surgical treatment in elderly patients with odontoid type II fractures [8,13,14,41,42]. In-hospital and early deaths did not occur. One-third of the patients died during the follow-up period, with an average time to death of 26.6 months. The overall survival was relatively high across the years, with a 1-year mortality of 13%, a 2-year mortality of 20%, and a 3-year mortality of 33%, but lower than in most published series [13,41,42,43].

In a survey to assess complications of operative and nonoperative treatment for odontoid type II fractures in patients over 80 years of age, Gembruch et al. [10] carried out a retrospective study based on 125 patients, of whom 98 had been treated surgically (65 anterior, 33 posterior, mean age 85.5 ± 4.2) and 27 conservatively (mean age 86.5 ± 4.3). According to the authors, the 90-day mortality was 27.8% in the surgical group and 20.0% in the nonsurgical group, with the difference between the two groups not being significant. [10].

One of the few prospective multicenter studies, published by Vaccaro et al. [42], included 159 elderly patients (65 years of age) with an odontoid type II fracture, among which 101 patients (63.5%) had received surgical treatment. The one-year mortality in the surgical group was not significantly lower than in the nonsurgical group (18% versus 28%). Complications occurred more often in the nonsurgical group at 36% versus 30%. Furthermore, a significantly lower nonunion rate was seen in the surgical group (5% versus 21%) [42]. Based on the same patient cohort in a multicenter prospective setting, Fehling et al. [14] could show a 2.92-fold higher risk of treatment failure when conservative strategies were chosen as compared to patients who had early surgery. Treatment failure was defined as decline in the Neck Disability Index by more than 9.5 points, major treatment-related complications, and death by any cause. Furthermore, the authors pointed out that initial conservative treatment, male sex, and older age were significantly associated with treatment failure [14].

Schroeder et al. [41] published a systematic review to address short- and long-term mortality and complications in surgically and nonsurgically treated patients with odontoid type II fractures. The authors concluded that patients who received surgical treatment, regardless of the type of surgery, had a decreased risk of short- and long-term mortality without an increased risk of complications [41]. Those results are consistent with the results of the previously reported, prospective, multicenter data [14,42].

Comparing the mortality rates of our study with rates in patients over 90 years of age who underwent surgical treatment for hip fractures, our study showed a lower one-year mortality, with 13% compared to 24%, and a higher postoperative walking ability with 93% [44].

Summarizing the literature, including two prospective multicenter studies [14,42], retrospective case series, and our results, there is a clear trend toward early surgical posterior stabilization in elderly and very elderly patients with unstable odontoid type II fractures. The rates of complications and mortality seem to be justifiable and outweigh the benefits of early mobilization and lower morbidity in most published studies [8,13,14,42]. On the other hand, there are still significant barriers to translating clinical findings into robust clinical practice guidelines due to the great heterogeneity of treatment effects and poor quality of the existing evidence, ranging from very low to low, which precludes us from drawing reliable conclusions.

### Limitations

The findings of this study should be interpreted within the scope of its limitations. Both the retrospective design and the small sample size might affect the generalizability of the results and can introduce selection and observer bias. Although the sample size is small, this is a rare population and, therefore, it would not be possible to include a larger sample size without having a national database. Furthermore, performance bias due to a procedure performed often at a high-volume center has to be taken into account. Additionally, a longer follow-up period may uncover other relevant information not captured in the current study. Finally, a lack of a conservatively treated control group is a major limitation of this study.

## 5. Conclusions

Our current study confirms that posterior stabilization of unstable odontoid type II fractures in patients over 90 years of age using CT-guided spinal navigation is a safe and effective procedure with a low complication rate. Despite an often high comorbidity rate in very elderly patients, we still recommend that surgery should be considered in patients over 90 years of age. The risks should be clearly discussed with the patient and relatives.

## Figures and Tables

**Table 1:** Inclusion/Exclusion Criteria.

Inclusion Criteria	Exclusion Criteria
Age ≥ 90 years	Congenital instability
Acute unstable type II odontoid fracture	Rheumatoid arthritis
Traumatic etiology	Instability caused by tumor
-	Spinal infection
-	Previous cervical surgery

**Table 2:** Patients’ characteristics.

Patient Number	SEX M = Male F = Female	Age (Years)	BMI ** (kg/m^2^)	ASA	AACCI ***
1	*M*	91	22	3	8
2	*F*	90	19	3	8
3	*F*	96	22.6	3	6
4	*F*	95	24.1	3	7
5	*F*	92	26	3	10
6	*F*	90	23.7	2	7
7	*F*	91	19	3	7
8	*M*	91	27.3	3	16
9	*F*	90	18.4	3	6
10	*F*	90	22.1	3	16
11	*F*	91	26.3	3	9
12	*F*	90	24.2	2	6
13	*F*	94	26.9	3	10
14	*F*	90	19.1	2	6
15	*F*	90	26	3	6
Mean (SD *)		**91.4 (1.9)**	**23.1(3.0)**	**2.9 (0.3)**	**9.1 (3.4)**

SD * = Standard Deviation, BMI ** = Body Mass Index, AACCI *** = age-adjusted Charlson Comorbidity Index.

**Table 3:** Clinical conditions for calculating the age-adjusted Charlson Comorbidity Index.

Comorbidity	Frequency (%)
Heart failure	6 (40%)
Diabetes mellitus	4 (27%)
Peripheral vascular disease	4 (27%)
Myocardial infarction	3 (20%)
Severe renal disease	3 (20%)
Tumor	2 (13%)
Cerebrovascular disease	2 (13%)
Chronic obstructive pulmonary disease	1 (7%)

**Table 4:** Major and minor complications occurred in the early postoperative phase. One patient had major and minor complications. All patients recovered uneventfully.

Major Complications	Frequency (%)	*n* = 1 (7%)
Pneumonic Sepsis	1 (7%)	
**Minor complications**	**Frequency (%)**	***n* = 4 (27%)**
Urinary tract infection	2 (13%)	
Delirium	1 (7%)	
Epistaxis	1 (7%)	

**Table 5:** Comparison of perioperative course and complication rates in patients over 90 years (Group A) to a retrospective analysis of patients between 65 and 89 years of age (Group B).

	Group A: Patients ≥ 90 (*n* = 15)	Group B: Patients 65–89 (*n* = 72)	Significance (*p* ≤ 0.05)
ASA * (1–4); Mean (SD)	2.9 (±0.3)	2.9 (±0.6)	NS
Postoperative ICU ** Stay (SD ***)	3.8 (±6.6)	1.9 (±2.1)	NS
**90-Day-Mortality (%)**	0 (0%)	2 (2.8%)	NS
**Postoperative** **neurological deficit (%)**	1 (6.7%)	2 (2.7%)	NS
**Minor complications**	4 (27%)	5 (6.9%)	NS
**Major complications**	1 (7%)	9 (12.5%)	NS

ASA *: American Society of Anesthesiologists. ICU **: Intensive Care Unit. SD ***: Standard deviation.

## Data Availability

The data presented in this study are available on request from the corresponding author.
